# Cystic intraductal papillary neoplasms with infiltrating carcinoma of the intrahepatic bile duct: A case report

**DOI:** 10.1097/MD.0000000000018758

**Published:** 2020-01-17

**Authors:** Zhoupeng Ma, Fang Zhao, Jiangfeng Pan, Guansheng Lin, Bingye Chen, Wenbing Fu

**Affiliations:** a Department of Radiology, Jinshan TCM-Integrated Hospital of Shanghai city; b Department of Radiology, The First Affiliated Hospital of Xiamen University, Fujian; c Department of Radiology, Affiliated Jinhua Hospital of Zhejiang University, Jinhua; d Department of Surgery, Jinshan TCM-Integrated Hospital of Shanghai city, Shanghai, P. R. China.

**Keywords:** cystic neoplasms, diagnosis, intrahepatic bile ducts, liver, papillary neoplasms, treatment

## Abstract

**Introduction:**

Intraductal papillary neoplasms of the bile duct (IPNB) is a kind of rare disorder with low incidence but high misdiagnosis due to untypical symptoms and non-specific laboratory indicators. Herein, we report a case of cystic type IPNB with infiltrating carcinoma of the intrahepatic bile duct presented as a single giant cystic mass of the liver.

**Patient concerns:**

A 51-year-old woman was admitted due to right upper abdominal discomfort for 10 months. Physical examination indicated no specific finding. Laboratory tests showed that serum total bilirubin and carcinoembryonic antigen level was mildly elevated. Ultrasonography, computed tomography (CT) and magnetic resonance imaging (MRI) of abdomen indicated a giant lobulated cystic lesion involving the left, right and the caudate lobes of liver. There were multiple small nodules of different sizes with papillary or coral reef-like pattern protruding into the cystic lumen from the inner wall.

**Diagnosis:**

The patient was diagnosed as malignant tumors of intrahepatic bile duct.

**Interventions:**

She received radical resection of the lesion by hepatectomy.

**Outcomes:**

The postoperative pathological examination revealed an IPNB with infiltrating carcinoma of the intrahepatic bile duct. This patient had an uneventful postoperative recovery and was discharged on day 21 post-surgery. Until 35 months after surgery, there is no recurrence or metastasis.

**Conclusion:**

The CT and MRI can show certain morphologic features including the segmental cystic dilatation of intrahepatic bile ducts and the pathological details of papillary tumors inside the lesion. Cystic IPNB with infiltrating carcinoma of the intrahepatic bile duct can be treated with surgery.

## 1 Introduction

Intraductal papillary neoplasms of the bile duct (IPNB) is a kind of rare tumor,^[1,2]^ which accounts for about 4% to 15% of bile duct tumors.^[3]^ It typically presents as multifocal lesions and exophytic growth pattern, and can involve any part of the biliary tree.^[4]^ For intrahepatic bile duct tumor, duct-ectatic type is common while cystic type is rare.^[2]^ Herein, we report a case of cystic type IPNB with infiltrating carcinoma of the intrahepatic bile duct, which presented as a single giant cystic mass of the liver. The imaging findings and pathological characteristics, clinical course and relevant literatures were reviewed and analyzed.

## 2 Case report

A 51-year-old previously healthy woman was admitted in January 2015 due to 10 months of right upper abdominal discomfort. On admission, serum total bilirubin level was 1.5 mg/dL and CEA level was 6.8ug/L. The routine blood, urine, and stool test and renal function revealed normal results. Physical examination showed normal findings. Ultrasonography indicated a giant cystic lesion in the liver, with interior inhomogeneous hypoecho, periphery inhomogeneous hyperecho, multiple nodules of the inner wall and dotted or striped blood flowing signals.

**Figure 1:**
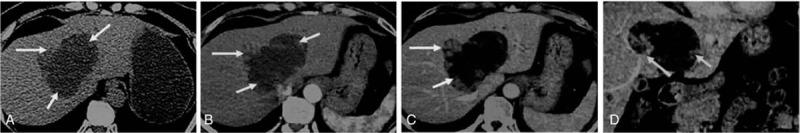
Computed tomography of a 51yr old female patient with intrahepatic ductal papillary neoplasm with infiltrating carcinoma. A giant lobulated cystic lesion inside liver was shown. (A) Unenhanced CT indicated that the fluid inside the lesion was hypodensity and there were multiple small nodules of the inner wall with hypo-density mildly relative to normal hepatic parenchyma (white arrows). (B) Arterial phase transaxial section indicated multiple nodules with obvious enhancement and indicated hyperdensity relative to normal hepatic parenchyma (white arrows). (C) Venous phase transaxial section indicated multiple nodules enhanced continuously but weaker than normal hepatic parenchyma, and fine septations inside the lesion with continuous enhancement (white arrows). (D) Venous-phase coronal reconstructive image indicated nodules with papillary (short arrow) or coralline pattern (long arrow). CT = computed tomography.

**Figure 2:**
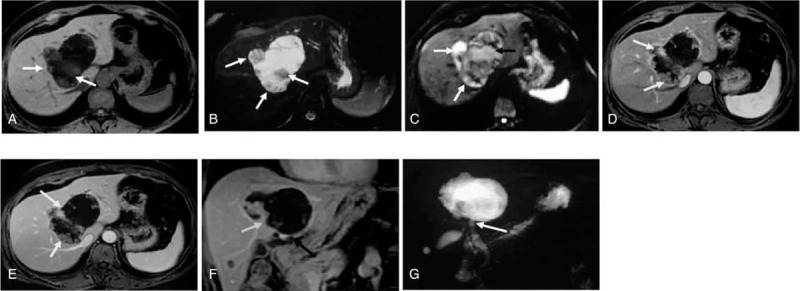
Magnetic resonance imaging of a 51yr old female patient with intrahepatic ductal papillary neoplasm with infiltrating carcinoma. (A) T1 transaxial section. (B) T2 transaxial section. Multiple nodules of inner wall showed T1 hypointense and T2 hyperintense relative to normal hepatic parenchyma (white arrows). Fluid inside the lesion showed signal as water. (C) DWI indicated multiple nodules (white arrows) and fluid inside the lesion (black arrow) with hyper-signal. (D) Arterial phase transaxial section. (E) Venous phase transaxial section. (F) Delayed-phase coronal section. The enhanced manifestation of multiple nodules was similar to CT (white arrows). The lower edge involved the hepatic capsule mildly (black arrow). (G) MRCP indicated a giant spherical lesion of hyper-signal communicated with the ectatic intrahepatic bile duct (white arrow). DWI = diffusion weighted images, MRCP = magnetic resonance cholangiopancreatography.

The patient underwent a triple-phase abdomen computed tomography (CT) scan including unenhanced, arterial, and venous phases. Unenhanced CT indicated a giant lobulated cystic lesion with heterogeneous cystic wall (thickness from 3 mm – 20 mm), which involved the left, right, and the caudate lobes, and the maximum diameter was 126 mm. The fluid inside the lesion showed hypodensity (CT value was from 9HU – 13HU). Multiple small nodules of different sizes (the maximum diameter was from 3 mm – 15 mm) and hypodensity (CT value from 19HU – 34HU) protruded into the cystic lumen from the inner wall (Fig. 1A). In enhanced CT, arterial phase images indicated multiple obviously enhanced hyperdensity nodules in the inner wall of cystic lesion (CT value from 68HU – 87HU) and fine septations inside the lesion with moderate enhancement were observed (Fig. 1B). In venous phase, multiple nodules of inner wall had continuous and inhomogeneous enhancement (CT value was from 82HU – 96HU) but weaker than normal hepatic parenchyma and showed inhomogeneous hypodensity relatively. Fine septations inside the lesion with moderately continuous enhancement were observed (Fig. 1C). Coronal reconstructive image of enhanced CT of venous phase indicated nodules of inner wall with papillary pattern predominantly, and a small proportion with shape of coral reefs. Fine fibrovascular cores were observed inside partial nodules. The lesion was confined inside the liver with the exception of involvement of the lower edge to the hepatic capsule, and adjacent structures including blood vessels and bile ducts were oppressed obviously but without infringement (Fig. 1D). No ascites in abdominal cavity or enlarged lymph node was observed in abdominal cavity or retroperitoneum in CT images. For further evaluation of the lesion, MRI was carried out subsequently. Compared to normal hepatic parenchyma, multiple nodules of inner wall showed inhomogeneous hyposignal intensity on unenhanced T1-weighted images (T1WI) (Fig. 2A) and mildly inhomogeneous hypersignal intensity on T2-weighted images (T2WI) (Fig. 2B). The fluid inside the lesion showed hypersignal as water. On diffusion weighted images (DWI), multiple nodules of inner wall showed inhomogeneous hypersignal intensity and fluid inside the lesion showed equal or hypersignal intensity (Fig. 2C). The enhanced manifestation of multiple nodules on enhanced magnetic resonance imaging (MRI) T1WI was similar to that on CT images. The outer wall of lesion was intact relatively and with obvious continuous enhancement (Fig. 2D–F). Magnetic resonance cholangiopancreatography (MRCP) indicated a giant spherical lesion of hypersignal intensity inside the liver, which communicated with the intrahepatic bile duct and the common bile duct, and the common bile duct showed mild dilatation (Fig. 2G). On the basis of these findings, a diagnosis of IPNB was suspected, and surgical resection was subsequently scheduled based on suspected malignancy.

**Figure 3:**
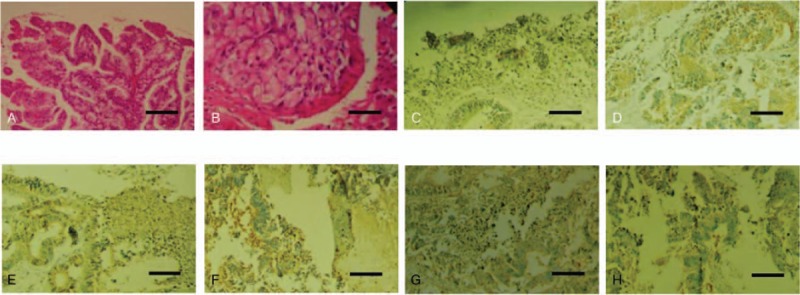
Pathology evaluation of a 51 yr old female patient with intrahepatic ductal papillary neoplasm with infiltrating carcinoma. (A) The intraductal components indicated papillary growth with fibrovascular cores (hematoxylin-eosin stain, scale bar = 400 μm). (B) Tumor cells showed obvious atypia and the base of tumors were confined in the epithelium of the bile duct, focal area of the ductal wall were invaded but without penetration (hematoxylin-eosin stain, scale bar = 50 μm). Immunohistochemical findings indicated positive expressions of Muc-5AC (C), Muc-6 (D), CK19 (E), CD34 (F), CK20 (G) and CK7 (H) (scale bar = 100 μm). CK = cell keratin, Muc = mucin core protein.

The patient underwent surgical resection at 11 days after admission. Exploratory operation revealed that the main part of lesion located in the left lobe. The right, caudate lobes of liver were locally involved and the lower edge of the lesion mildly involved the hepatic capsule. Hepatectomy was performed and the lesion was completely resected. Postoperative macropathology found that the resected specimen was a giant multilobulated cystic mass with heterogeneous cystic wall (wall thickness from 3 mm –18 mm) and soft texture, and was 118 mm × 116 mm × 65 mm in size. The cystic lumen was filled with yellowish mucin-like fluid and the section of the cyst wall was greyish red. Multiple nodules of different sizes protruded into the cystic lumen from the inner wall. Microscopically, the intraductal components showed papillary growth with fibrovascular cores (Fig. 3A). The surface of papillary tumors was covered with intestinal epithelium. Tumor cells showed abundant cytoplasm and large nucleus. Vessels in local interstitium were abundant. The bases of tumors were confined in the epithelium of the bile duct, focal ductal interstitium were invaded but without penetration and the hepatic parenchyma was not invaded (Fig. 3B). Immunohistochemical analysis indicated positive expressions of mucin core proteins (Muc)-5AC (Fig. 3C), Muc-6 (Fig. 3D), cell keratin (CK)19 (Fig. 3E), CD34 (Fig. 3F), CK20 (Fig. 3G) and CK7 (Fig. 3H), while negative expressions of arginase-1 and Muc-2. The final pathological diagnosis was IPNB with infiltrating carcinoma of the intrahepatic bile duct (intestinal type). The patient had an uneventful recovery and was discharged on day 21 post-surgery, and did not receive any other treatments after surgery. At 35 months of postoperative follow-up, no recurrence or metastasis was observed by ultrasonography.

## 3 Discussion

IPNB was first proposed as a distinct entity in the 2010 revision of the World Health Organization classification for liver and intrahepatic bile duct tumors.^[5]^ It is a recognized precursor of invasive carcinoma frequently arising during a reactive change in chronic biliary tract disease. However, pathogenesis and natural history of IPNB is still unclear.^[6]^ Clonorchiasis and hepatolithiasis has been implicated as causes of IPNB.^[7,8]^ Clinical manifestations of IPNB include epigastric discomfort, biliary colic, jaundice or intermittent fever.^[2,4]^ In this case, the main complaint was right upper abdominal discomfort. In absence of typical liver related presentations such as biliary colic, jaundice or intermittent fever, the diagnosis and treatment of this patient was delayed.

IPNB can develop anywhere along the biliary tree including both intrahepatic and extrahepatic bile ducts.^[1]^ Intrahepatic IPNB is the most common type, which includes duct-ectatic type and cystic type, and the former accounts for the vast majority.^[2,8]^ IPNB is characterized by a spectrum of diseases ranging from low-grade intraepithelial neoplasia to invasive carcinoma, including adenoma, carcinoma-in-situ and invasive carcinoma.^[9]^ Some morphologic features of IPNB have been identified, such as diffuse or segmental ductal dilatation and the appearance of an intraductal growing mass. Nearly one-third of IPNB cases are associated with macroscopic mucin hypersecretion, therefore, bile duct dilation is often observed.^[1,2]^ Microscopically, IPNB is composed of papillary fronds with fine fibrovascular cores. Neoplastic epithelial cells display a spectrum of cytoarchitectural atypia ranging from none to borderline, and also can be associated with invasive carcinoma.^[2]^ According to the microscopic appearance, IPNBs are classified into 4 epithelial subtypes, including pancreatobiliary, intestinal, gastric, and oncocytic types.^[7,10]^ The most frequent subtype is pancreatobiliary, followed by intestinal IPNB. As pancreatobiliary or the intestinal type is commonly associated with high-grade intraepithelial neoplasia, most of IPNB cases are pathologically diagnosed as intraductal papillary neoplasm (IPN) with high-grade intraepithelial neoplasia or IPN with an associated invasive carcinoma.^[11]^ The histologic types of intestinal IPNB usually refer to mucinous carcinoma.^[10,11]^ In the present case, the final pathological diagnosis was IPNB with invasive carcinoma (intestinal type) based on microscopic findings.

Preoperative misdiagnosis of IPNB is frequent in clinical practice due to low incidence, lack of specific tumor markers and unclear pathogenesis.^[4,12]^ Fortunately, with the improvement of imaging equipment and diagnostic technology, the early diagnosis rate of IPNB is increasing. Imaging including CT and MRI can show certain pathological characteristics of IPNB. The most common imaging findings of IPNB include dilated bile ducts, intraluminal mucin, and lesions protruding into the lumen. On unenhanced CT, papillary tumor tissues, fine septations and mucus secreted by tumor tissue usually show hypo-density, but the density of mucus is generally higher than that of water.^[9,13]^ On unenhanced MRI, structures mentioned above usually show hyposignal intensity on T1WI while hypersignal intensity relatively on T2WI and DWI.^[9]^ On enhanced scan of CT and MRI, the enhancement of papillary tumor tissues is always more obvious in arterial phase and show hyperdensity/signal compared to normal hepatic parenchyma, especially for invasive carcinoma, and fine fibrovascular cores inside tumors may be clearly displayed in some cases.^[12,13]^ While in venous phase, the enhancement is continuous but weaker than normal hepatic parenchyma and show hypodensity/signal relatively, and the enhancement usually declined in delayed phase.^[14]^ On MRCP, most lesions communicated with the biliary structures.^[15]^ Moreover, other abdominal lesions including celiac and retroperitoneal metastases can be observed clearly. In the present case, it is quite rare that the IPNB of intrahepatic duct presented as a single giant cystic mass, but multiple tumors inside the lesion showed papillary or coralline appearance and with obvious enhancement in arterial phase, which showed typical characteristics of IPNB.

Differential diagnosis:

(1) hepatic mucinous cystic neoplasm. Most hepatic mucinous cystic neoplasms occur in female and young population, and manifest as solitary, polycystic lesion with larger cystic cavity. In addition, there was no communication between lesion and bile ducts and calcification of cystic wall can be observed generally.^[16]^
(2) Intrahepatic cholangiocarcinoma. Intrahepatic cholangiocarcinoma is the most common malignant tumor of intrahepatic bile duct and has a higher incidence in elderly male population.^[17]^ On CT and MRI, the tumors show hypodensity/signal generally and delayed enhancement on enhanced images. Moreover, dilatation of upstream bile duct can be observed whereas the downstream bile duct of lesion is normal generally.^[18]^ Furthermore, lymph node or distant metastasis is more frequent.^[4,17]^
(3) Simple hepatic cyst: The cyst wall of simple hepatic cyst is thin and smooth, and there is no nodule inside the lesion or communication between lesion and intrahepatic bile duct. Some cystic IPNBs lack obvious papillary structures and have similar imaging findings as simple hepatic cysts.^[9]^ For these cases, ^18^F-fluorodeoxy -glucose positron emission tomography may demonstrate an FDG avid area in the cyst wall, which corresponds to the tumor tissue.^[14]^

Lymph node metastasis or distant metastasis is much less in patients with IPNB than in those with conventional cholangiocarcinoma, and surgical resection is considered as the optimal treatment modality for IPNB.^[4,12,18]^ Hepatectomy is suitable for lesions of the intrahepatic bile duct and porta hepatis, while local excision of the biliary tract is applicable for lesions of the middle part of the extrahepatic bile duct, and pancreaticoduodenectomy is suitable for distal extrahepatic bile duct tumors.^[4]^ If the intrahepatic lesion is too large to be completely resected, preoperative portal vein embolization may be carried out to induce hypertrophy of the remnant liver.^[14]^ In cases with bilateral involvement or with terminal liver disease that precludes a partial resection, liver transplantation has been shown to obtain good survival rates.^[19]^ Adjuvant therapies including chemotherapy, percutaneous transhepatic biliary drainage, percutaneous cholangioscopic laser ablation and iridium-192 intraluminal therapy are recommended when major surgery is not indicated.^[20]^

Although approximately 40% to 80% of respectable IPNBs contain invasive components, the prognosis is more satisfactory than that of other cholangiocarcinomas.^[12]^ Moreover, there is no correlation between clinical presentation and survival.^[2,7,9]^ The patient in our study underwent a hepatectomy and had an uneventful recovery after operation without recurrence or metastasis during 35 months of follow up.

In conclusion, cystic type IPNB of the intrahepatic duct is fairly rare. The CT and MRI can show certain morphologic features including the segmental cystic dilatation of intrahepatic bile ducts and the pathological details of papillary tumors inside the lesion. Surgical excision is a reliable and effective treatment for most cases, which also can bring a relatively well prognosis for such patients.

## Author contributions

**Conceptualization:** Jiangfeng Pan.

**Data curation:** Zhoupeng Ma, Fang Zhao.

**Investigation:** Bingye Chen.

**Methodology:** Guansheng Lin.

**Resources:** Jiangfeng Pan.

**Software:** Wenbing Fu.

**Writing – original draft:** Zhoupeng Ma, Fang Zhao.

**Writing – review and editing:** Zhoupeng Ma.
